# The Potential and Limitations of the MinION/Yenos Platform for miRNA-Enabled Early Cancer Detection

**DOI:** 10.3390/ijms26083822

**Published:** 2025-04-17

**Authors:** Aleena Rafiq, Anastassia Kanavarioti

**Affiliations:** Yenos Analytical LLC, El Dorado Hills, CA 95762, USA

**Keywords:** miRNA, cancer biomarker, nanopores, MinION, osmylated nucleic acids, probes, total RNA isolation, liquid biopsies, multiple-cancer early detection (MCED)

## Abstract

The 2024 Nobel Prize in Physiology or Medicine was awarded to the pioneers who reported that microRNAs (miRNAs) regulate and direct the switch between physiological and pathological pathways via their over- or underexpression. The discovery changed the medical landscape and there are many completed and on-going clinical studies based on miRNAs. MiRNAs occur at the femtomolar level in biological fluids and are typically quantified using amplification-based techniques. Experimental nanopores have illustrated potential for trace analysis including amplification-free miRNA quantification. We repurposed the MinION, the only commercially available nanopore array device, and developed unique probes and protocols to detect and measure miRNA copies in blood and urine. Here, we report that miRNA copies are proportional to the total RNA isolated from the biospecimen, and that three known miRNA cancer biomarkers, i.e., miR-21, miR-375, and miR-141, were more than 1.5-fold overexpressed in blood samples from breast, ovarian, prostate, pancreatic, lung, and colorectal cancer patients compared to healthy patients. In these cancer samples, miR-15b was not overexpressed, in agreement with earlier studies. In contrast to literature reports, sample variability was undetectable in this study. The potential and limitations of this ready-to-use MinION/Yenos platform for multiple-cancer early detection (MCED) using blood or urine are discussed.

## 1. Introduction

Cancer screening has been a major research front for decades [1,2,3,4]. The classical circulating blood biomarkers for cancer, such as prostate-specific antigen (PSA), carcinoembryonic antigen (CEA), cancer antigen 125 (CA125), alpha-fetoprotein (AFP), etc., are neither sensitive nor specific. These tests are often inconclusive and not recommended for population screening [3,4]. Several cancer indications, such as pancreatic, typically shows no early symptoms and late stage may be inoperable [5]. There is a worldwide push to include liquid biopsies as companion tests to help medical professionals cover the “grey” area of the current assays [6,7,8,9,10,11].

By 2001, several lines of research had led to the discovery of miRNAs and the proposition that they regulate the post-transcriptional expression of proteins and the switch from physiological to disease pathways [12,13]. miRNAs are single-stranded (ss) RNAs around 22-nucleotide long and are stable in biological fluids, which renders them suitable biomarkers [14,15]. Currently there are over 2500 human miRNAs known [16,17], and they are the subject of over 80,000 medical studies which have associated abnormal expression of selected miRNAs with cancer onset, progression, prognosis, and metastasis [18,19,20].

Most of the 80,000 “miRNA cancer” studies have concluded that a single miRNA or an miRNA panel may serve as a cancer biomarker [18,19,20]. Typically, the data exhibit high variability. Data from the cancer samples overlap with data from the healthy samples. Therefore, investigators report the mean, instead of the average/standard deviation of the measurements and compare the mean of the cancer samples to the mean of the healthy samples [21,22,23,24,25]. As an example, see in Ref. [21] Fig. 2c for miR-141 data spread and overlap). Further statistical analysis yields a Receiver Operating Characteristic (ROC) curve and a score of how well the specific miRNA, or miRNA panel, prognoses cancer progression. A good score may be satisfactory in the case of a drug that treats a life-threatening disease, but a good score is not sufficient to diagnose cancer in an asymptomatic individual. This “data spread” may be partially responsible for the absence of FDA-approved miRNA-based screening tests and for delaying the development of anti-miRNA-based therapeutics [19,20].

The overlap of miRNA data between cancer and healthy samples and the quantitative disagreement among studies have been attributed to differences in biospecimen collection methods, study protocols, choice of reference and normalization, analytical method, population variation, age, sex, ethnicity, disease stage, etc. [21,22,23,24,25]. This uncertainty led to questioning the usefulness of miRNAs as liquid biopsy biomarkers.

The concentration of miRNAs in blood is in the low femtomolar (fM) range, which is a billion-fold less than the micromolar (μM) range required by typical UV-Vis analytical tools. Current methods for assessing miRNA abundance depend mostly on amplification using Polymerase Chain Reaction (PCR) and include small-RNA sequencing, reverse transcription-quantitative PCR (RT–PCR), droplet digital PCR (ddPCR), microarray hybridization, and Northern Blot [21,22,23,24,25]. Although identification works well with these tools, the quantification accuracy and the choice of normalization have been questioned and may be partially responsible for the “data spread”.

**Figure 1:**
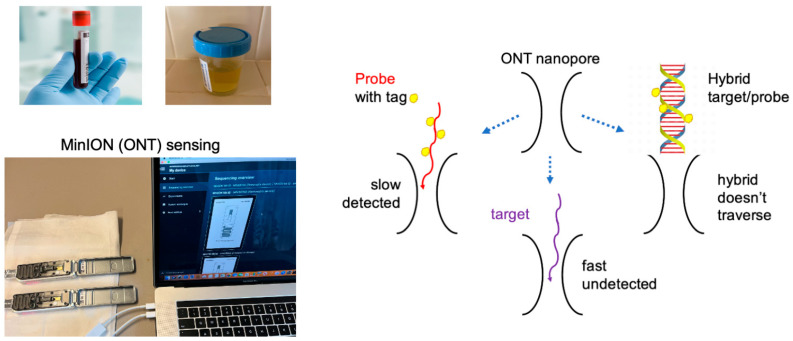
From [29,31,32]. A graphical abstract of the processes involved in the miRNA measurement using the MinION/Yenos platform. Left, top: Collection of the biospecimen, blood, or urine, followed by total RNA isolation using a commercial kit and measurement of total RNA in the isolate using a nanodrop spectrophotometer. Left, bottom: Mixing an aliquot from the RNA isolate with an aliquot of the probe complementary to the target miRNA (Materials), adding ONT buffer and conducting a MinION unassisted ion conductance experiment (two experiments running simultaneously, shown here). The experiment measures the ion current (*I*) in picoamperes (pA) as a function of time (t) in milliseconds (ms). *I* is a constant at *Io*, which is the open nanopore ion current (*Io*). When a single molecule traverses the nanopore, *Io* is reduced to a new value, *Ir*, because the molecule occupies the space that would have been occupied by the electrolyte that produces *Io*. Ion current reduction (dip in this platform) lasts for a time, τ, and produces an event that is read by software (*OsBp_detect*, see Section 4). The analysis determines whether the free probe is in excess and detected (left on the scheme above) or if the probe is not detected because it is hybridized with the target (right on the scheme above). Notably, RNAs, including the target miRNA, traverse much faster than the probes, and they are mostly missed (bottom on the scheme above) due to the relatively slow acquisition rate of this platform. A probe is an oligodeoxynucleotide complementary to the target miRNA, loaded with osmium tags (see Section 4, Yenos probes), optimized for hybridization with the target and for detection by the nanopores [32].

We hypothesized that an amplification-free approach to quantitating miRNAs may reduce sample variability. In contrast to traditional analytical tools which require amplification, nanopores appear well suited for trace measurements. Numerous studies document the use of experimental nanopores to detect charged chemical and biological components including miRNAs ([26,27,28], and references therein). In 2014, Oxford Nanopore Technologies (ONT) introduced a nanopore array-based analytical platform, the MinION, designed to sequence long DNA/RNA. Sequencing is enabled by an enzyme-assisted, slowed-down translocation of a nucleic acid single strand (ss) via a pore. Currently, the MinION is the only commercially available nanopore array device. We repurposed this device for unassisted, direct detection/quantification (called sensing) and showed proof-of-principle with ss short nucleic acids (Figure 1 and [29,30]). We then focused on miRNAs and used total RNA isolated from a biospecimen as the source. A probe was developed with a general design to facilitate pore translocation and made unique by incorporating a sequence complementary to the target miRNA [29,31]. A sample is prepared by mixing an aliquot from the total RNA isolate and the probe complementary to the target miRNA and the mixture is loaded onto the MinION/flow cell that houses the nanopores. During the experiment, the free probe molecules translocate via the pores and are selectively detected. The hybrid formed between the target and the probe is too big and does not fit via the pore. Unhybridized miRNAs and other RNA molecules translocate much faster than our proprietary probes and are mostly missed by the relatively slow data acquisition rate of the MinION (see Scheme in Figure 1 and [31,32]). Quantitation is enabled using quality probes of known concentration (see Section 4).

**Figure 2:**
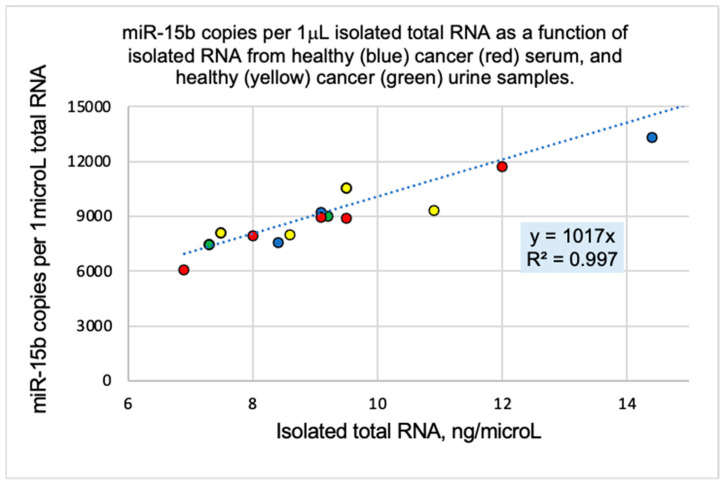
miR-15b copies are proportional to the total RNA content of the isolate obtained from the biospecimen and are independent of the specimen (blood or urine), RNA collection kit, age, sex, ethnicity, and the presence/absence of cancer. Half of the data are reported in [31] and the rest in [32] and are plotted here together.

**Figure 3:**
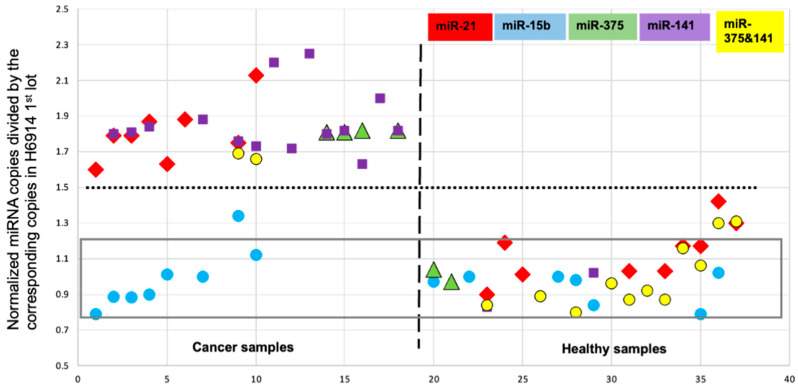
From [32]. miRNA copies were normalized to the same RNA content (16 ng/mL) and then divided by the corresponding miRNA copy number in the reference H6914 (combined serum, healthy men assigned as 1.0 HL) to obtain x-fold HL (HL stands for healthy level). In serum and urine samples from cancer patients, miR-21, miR-375, and miR-141 measured at 1.83 HL (RSD = 0.09), and in healthy urine samples at 1.04 HL (RSD = 0.17) with zero data overlap (*p*-value 1.6 × 10^−22^). The threshold between cancer and healthy samples is at 1.5 HL (dotted line). In contrast to the other three miRNAs, and the two-miRNA combination 375 and 141, miR-15b is not overexpressed in the cancer samples.

A commercial combined serum product was used as the reference to optimize probe design, guide the development of the experimental protocol, and facilitate the interpretation of the data (see Section 4, cat no H6914). This technology is low-throughput and yields absolute miRNA copies per 1 μL of isolated total RNA. In an earlier study, we discovered that miRNA-15b copies were proportional to the total RNA content of the biospecimen (Figure 2 and [31,32]). This led us to normalize measured copies for every tested miRNA to the same total RNA content (16.0 ng/μL). Besides miR-15b, we measured several other miRNAs in the above reference and used these reference copy numbers to normalize measured miRNAs in all tested samples. These two normalizations (total RNA content and reference miRNA copy number) enabled the inclusion of all the data in one figure (Figure 3 and [32]). Figure 3 includes serum and urine samples, samples from patients with breast, prostate, or pancreatic cancer, and subjects that differ in age, sex, and ethnicity. Figure 3 illustrates x-fold (x HL) expression of a certain miRNA in a sample compared to the healthy reference (HL).

Despite the broad diversity in this cohort, the data appear to yield two groups only (Figure 3 and [32]). The first group includes all the healthy samples regardless of target miRNA and the data obtained with the cancer samples targeting miR-15b, confirming that miR-15b is not a cancer biomarker [14]. The second group includes the data from the cancer samples targeting miR-21, miR-375, and miR-141, confirming that these three miRNAs are cancer biomarkers [14,33,34]. In contrast to literature reports where sample variability is evident, the data in Figure 3 are described by an average value of 1.04 HL (HL stands for healthy level) for the first group and an average value of 1.83 HL for the second group. Figure 3 illustrates that each of the three miRNAs may serve as a single cancer biomarker, with zero data overlap between cancer and healthy samples. The data further demonstrate an apparent threshold of 1.5 HL, the same for all three miRNAs and for all three cancer indications [32]. To the best of our knowledge, this is the first time such statistically unlikely “coincidence” has been reported. To probe these observations and, perhaps, extend these findings to additional cancer indications, we report here on experiments that target the 1.5 HL threshold using new samples from treatment-naïve patients with breast, prostate, pancreatic, ovarian, lung, and colorectal cancer.

## 2. Results

Based on the earlier findings with breast, prostate, and pancreatic cancers, we postulated that miR-15b, miR-21, miR-375, and miR-141 may follow the observed pattern (Figure 3, 1.5 HL threshold) in other cancers. We chose to evaluate the postulate by testing with ovarian, colorectal (CRC), and lung cancer as additional cancer indications. In this study, new probes for each miRNA (miR-15b, miR-21, miR-375 and miR-141) were manufactured based on published methods [32] and used for all the nanopore experiments reported here. Two new lots of the reference combined serum H6914 were purchased and retested (see Section 4). New serum samples were procured from stage I/II treatment-naïve patients with breast, prostate, pancreatic, ovarian, colorectal (CRC), and lung cancers. Two healthy subjects provided urine samples, as part of an IRB-reviewed study (see Section 4). Total RNA was isolated from serum or urine using the appropriate commercial isolation kits (see Section 4). All these samples, a total of 26, were exploited as a training set to further optimize the experimental protocol of the nanopore experiments and the data analysis methodology. Selected experiments were conducted with the reference H6914 and the healthy urine samples and confirmed the earlier measured miRNA copy numbers [see 1st page in Supplementary Materials (entries in red)]. Selected experiments were also conducted with one CRC and two lung cancer samples and showed that cancer miRNA biomarkers are about 1.8-fold overexpressed and that miR-15b is not overexpressed [see 1st page in Supplementary Materials (entries in red)].

The above selected experiments were consistent with our postulate and led us to forgo measuring miRNA copy numbers in all the samples. Instead, we designed experiments to target the 1.5-fold healthy level (HL) which is the observed zero data overlap threshold between cancer and healthy samples (Figure 3). This strategy reduced the number of experiments by more than half. Specifically, the experiments were designed to test whether a certain miRNA in a sample measures above or below 1.5 HL. As the miRNA copy number (1.0 HL), here, we used posted data with miR-15b = 17,710, miR-21 = 10,494, miR-375 = 9240, and miR-141 = 6096 per 1 μL of total RNA or the equivalent of 2 μL of serum [31]. Notably, the serum isolation kit yields 100 μL of total RNA from 200 μL of serum, and the urine isolation kit yields 50 μL from 5 mL of urine (Section 4.1), which practically equalizes the miRNA levels between these two specimens. Based on the earlier findings, miR-15b must measure below the 1.5 HL threshold for all samples, whereas miR-21, miR-375, and miR-141 must measure above 1.5 HL in the cancer samples but below 1.5 HL in the healthy samples [32]. Table 1 summarizes the results and the statistics for the “true” result for each miRNA. The Supplementary Materials contain the graphical result of the test for each entry in Table 1. Since the objective of this study was to optimize the protocol and the analysis, the data obtained are not all optimal. Presumably, the optimized protocol, as described below, may produce statistics equal or better than those obtained in this study.

It is worth noting that Table 1 includes data from a cohort of over 120 samples that differ in 16 known variables: 6 cancer indications, 4 distinct miRNAs, biospecimen (serum or urine), cancer/no cancer, collection kit, age, sex, and ethnicity. Remarkably, the data in Table 1 reduce the 16 known variables to 3: (i) total RNA content isolated from the biospecimen normalized to the same RNA content of 16 ng/μL, (ii) the presence/absence of cancer in the sample, and (iii) whether the target miRNA is a cancer biomarker or not.

The accuracy, sensitivity, and specificity of a test may be easily mixed up. With this novel technology, the accuracy of an miRNA measurement, as described above, is defined by the user/protocol, and at least three Yenos tests are required to determine and confirm an miRNA copy number and its accuracy. Preliminary data suggest that both the sensitivity and specificity of this technology are 100% [32]. This is equivalent to the observation of zero data overlap between cancer and healthy samples, admittedly in a small-size cohort. In the latter, miRNA copy numbers were measured, sometimes conducting more than three tests per determination. In the current study, there was only one Yenos test conducted, and no miRNA copy number was determined for most of the samples (see exceptions in the 1st page of the Supplemental Materials). Since this cohort served as a training set, we can calculate the success rate or true score of a single Yenos test, which was found to, at best, 97%. This conclusion is not in disagreement with the reported practically 100% sensitivity and specificity of miRNA copy number determination [32]. The important question for which it is too early to give an answer to is whether miRNA copy numbers of the above tested miRNA biomarkers are truly independent of the many variables which until now were believed to rationalize sample variability.

## 3. Discussion

### 3.1. Comparison of the MinION’s Sequencing vs. Sensing to Detect/Quantify Short SS Nucleic Acids

Recently, the MinION’s sequencing protocol was explored for multiplexed biomarker detection [35]. The authors reported an miRNA detection limit (DL) of about 50 pM, which happens to be too high for liquid biopsy testing. For example, miR-16, one of the most abundant miRNAs in blood, measures about 170 fM [31], about 300 times less than the above DL. One of the least abundant miRNAs is miR-141, which was recently quantified accurately at about 5 fM using our sensing approach [31,32]. In addition, 5 fM compared to 50 pM represents a better than 10,000 sensitivity improvement, illustrating the suitability of the MinION/Yenos platform for quantifying miRNAs in liquid biopsies.

### 3.2. The Limitations of the MinION/Yenos Platform for Cancer Screening and Sensing

Analytical assays report a range for which they are useful and a DL; this new platform differs. It uses equally well serum or urine, even though urine is about 50 times more dilute in nucleic acid components compared to serum. As mentioned earlier, it is the commercial kit for isolation of total RNA from urine that yields total RNA comparable to what we obtained from serum using the Monarch kit. Preliminary experiments suggest a range of 1 fM < miRNA < 170 fM per 1 μL total RNA, and we have not established a lower or an upper limit. This relatively broad range for a nanopore-based assay is somewhat misleading. The range is obtained by controlling sample composition. Probes can be used at a higher or lower concentration and the total RNA isolate may be diluted or concentrated. Our development work has led us to an optimal level of probe molecules that is detectable over the current noise of the MinION (see Section 4), although a lower noise level may be obtainable with ONT involvement.

Since the proposed protocol uses a “buffer only” experiment between samples, contamination is at a minimal and samples from distinct subjects can be analyzed in the same flow cell. However, the ONT proprietary flow cells are not resilient. This may be attributed to the proteinic nature of the nanopores and the fact that a nanopore is composed of seven to eight peptide strands that, to our knowledge, are not covalently attached to each other. If this is true, it is easy to see why the relatively fast translocation of millions of molecules lessens the pore’s lifetime. The friability of the nanopores reduces the number of active pores with use and, in turn, reduces the number of translocating molecules. The latter necessitates that a control/buffer experiment is conducted ahead of a new sample. Notably, the ONT buffer is promoted for sequencing and may be less optimal for sensing (see Section 4). Since the buffer is proprietary, ONT is best equipped to optimize it for sensing. Another issue is that the flow cell is outfitted with 2024 nanopores but there are only 512 detection channels. Perhaps randomly, different pores are selected every time a new experiment is initiated. In our experience, nanopores exhibit substantial differences among them and changing from one group to another reduces reproducibility. To improve reproducibility, the optimized protocol recommends two 45 min runs with the buffer and then two 45 min runs with the sample. Equivalency between detection channels and nanopores may be a better strategy for sensing. In addition to these limitations, the high cost of a flow cell is currently an issue and may prohibit researchers from exploring the MinION/Yenos platform. This platform is ready-to-use and requires only minimal infrastructure and junior scientist-level training. It could be implemented at a point-of-care facility (POC). However, ready-to-use does not mean user-friendly. ONT supports sequencing and not sensing. The ONT software MINKNOW could be adapted to sensing for the interested user, screens could be modified, and *OsBp-detect* software (version 1.22.0) [30] could be incorporated. An algorithm to interpret the data should be included. Data interpretation is now carried out manually; it is time-consuming and mimics an “assembly line” of transferring files, running programs, graphing, calculating, etc. Most, if not all, of these issues may be solvable with ONT collaboration/involvement.

One of the serious limitations of this technology is also its biggest advantage. The assay uses a bracketing approach, namely two Yenos tests are conducted, a silencing and a detection test, and these two tests are designed to differ by about 40% in one of the components so that the miRNA measured value is protocol-defined to be accurate to about ±20%. It turns out that to assess an over- or underexpression of 1.8-fold, the measurement must have a ±25% accuracy (see Table 1A in [32]). This is the strength of this assay. Nevertheless, a bracketing experimental approach is useful in confirming a known value and not determining an unknown value. Two to three Yenos tests are required for a confirmation measurement. Three Yenos tests require the full use of a flow cell which currently costs USD 600, not counting the cost of the other supplies and the cost of labor/facilities. To determine the copy number of an unknown miRNA, many more Yenos tests are needed, perhaps starting from a range of 0.3–3.0 HL and designing experiments inside this range until a set of detection/silencing that is only 40% off in one of the components is reached. Despite the remarkable accuracy, such low-throughput technology will only be useful in validation cases, unless the 1.5 HL threshold strategy which worked out well for the samples tested in Table 1 ends up being more broadly applicable.

A major step forward will be achieved when a comparable device is equipped with a flow cell made of solid-state nanopores which should be more resilient than the proteinic nanopores [36]. If the nanopores are wide enough to fit a single RNA strand but narrow enough to prohibit translocation of double-stranded DNA/RNA, our probes may be usable without modification. The cost of a flow cell with a solid-state nanopore array may be much lower than the current flow cell cost.

### 3.3. The Potential of the MinION/Yenos Platform for Cancer Screening

The data in Table 1 were collected with slightly modified protocols in search for an optimal one. The optimal protocol incorporates two 45 min experiments with a buffer and two 45 min experiments with the sample of interest; these four experiments represent a single test, the Yenos test. Both the protocol to acquire the data and the methodology to obtain the result are detailed under Section 4. The sample contains a known amount of probe copies equal to 1.5-fold (1.5 HL) of the corresponding miRNA copies in the reference H6914. The test has a binary result. When the probe copies are more than the target miRNA, the result is the detection of the free probe. When the probe copies are less that the target miRNA copies, then the result is no probe detection or silencing (see Scheme in Figure 1). Practically speaking, the later experiment appears more like a buffer experiment. The experimentation testing the 1.5 HL threshold is highly limited but apparently worked with this cohort (Table 1). It was carried out to reduce the number of experiments. The observation that 16 variables are reduced to 3 (see discussion of the table) leads to the conjecture that these three miRNA cancer biomarkers are cancer-agnostic, overexpressed to the same level, and independent of demographics. This is an unprecedented statement but with implications that warrant further exploration. It is conceivable that such observations were made because the technology uses a suitable normalization (the total RNA of the sample) and a bracketing experimental protocol that allows one to define a priori what accuracy is acceptable for the specific measurement (set at about ±25% in our studies). Notably, the 1.8-fold overexpression of miRNA cancer biomarkers observed in our studies necessitates an accuracy better than ±25% to yield zero data overlap (Table 1A in reference [32]). A lesser accuracy may rationalize the data overlap observed with other analytical techniques.

The sensitivity/specificity of the optimized protocol is believed to be around 97%, regardless of whether the Yenos test yields a detection or a silencing result. The cohort in Table 1, represents a small study, and therefore, the findings need to be confirmed in a larger-sized cohort. Assuming a validated 97% true result, a single test per subject will yield 3 false results out of 100. Two tests per subject will give 9 false results in a group of 10,000, and three tests will give 27 false results in a group of 1,000,000 subjects. These calculations presume that assignment will be made if all tests agree. Statistically with three tests at a 97% true result, 8.7% will show agreement between two tests, while the third test will differ. One way to confirm the result in such a case is to conduct a fourth test with the 8.7% population.

Currently, one can conduct three such tests—each test consists of four 45 min nanopore experiments—on a single flow cell. If the optimized protocol yields a 97% “true” assignment (Table 1), then the MinION/Yenos platform may turn out to be useful for MCED testing as is. The issue with most of the MCED tests in development is that they look for cancer evidence in the form of tumor DNA fragments. This approach informs whether a subject has detectable cancer. The miRNA hypothesis is leaning on the premise that aberrant expression precedes the onset of cancer. If this is valid and the selected miRNAs are the tiny regulators [12], then controlling the regulator(s) may not be any different than controlling high blood pressure so than one does not die from a heart attack at some point. There is optimism that controlling miRNA aberrant expression may eradicate cancer and other diseases.

## 4. Materials and Methods

### 4.1. Human Samples

Human serum from the USA, isolated via sterile filtration from male AB-clotted whole blood (H6914), was purchased from Sigma-Aldrich (St. Louis, MO, USA, Millipore now) and has been used as the reference for all our work. Different lots have been purchased and tested over the last 5 years and comparable results were obtained. In all of the studies, miRNA copies at 1.0 HL are the miRNA copies determined in the first such study [31]. New lots used here are SLCN9218 (SQ H6914) and 0000305265 (H6914 2025 or H6914 12.1 ng/μL). Serum samples purchased from Discovery Life Sciences (DLS, Huntsville, AL, USA) and Tissue for Research (Accio Biobank online, Suffolk, UK) were collected from informed consenting individuals under the IRB/EC protocol. The selection of these samples from a large depository included both male and female donors, if applicable, and one each of African American, Hispanic, or White ethnicity. Samples were collected from newly diagnosed, naïve patients ahead of treatment. The demographics of the patients with cancer who provided their specimens are listed in Table S1 (Supplementary Materials). The project to include urine samples collected by themselves at home and sent by FedEx overnight to our facilities from consenting healthy subjects was reviewed by the Advarra Investigational Review Board (IRB). The protocol and consent form were reviewed, modified, and approved by the Advarra IRB on 15 November 2023. Protocol: Yenos Analytical LLC-02. Quantification of selected microRNAs in the urine of healthy individuals (Pro00074065) was performed with continued review approval on 25 October 2024 (CR00599062). Donors of urine samples reviewed and signed an informed consent form. For the isolation of total RNA from serum, we used the Monarch T2010S Kit from New England Biolabs. For the isolation of total RNA from urine, the Norgen Biotek Corp. No. 29,600 kit was used with 5 mL urine sample. Kits were used according to the manufacturers’ instructions.

### 4.2. Oligos, Probes, and Other Reagents

The only ONT kit used for the experiments reported here was the Flow Cell Priming Kit XL (EXP-FLP004-XL), called here ONT flush buffer or ONT buffer. The ONT buffer is proprietary, provides the necessary electrolytes, and must represent more than 80% of the 80–85 μL sample volume. Custom-made DNA oligos and 2’-OMe-oligos synthesized at the 0.2 mmole scale and purified by HPLC or PAGE by the manufacturer were purchased from Integrated DNA Technologies (IDT). Oligos (sequences in Table 2) were diluted with Ambion nuclease-free water, untreated with DEPC, typically to 100 or 200 μM stock solutions, and stored at −20 °C. Oligo purity was confirmed to be >85% by in-house HPLC analysis [37]. Osmylated oligos were manufactured in house, based on published methods at a concentration of approximately 20 μM and purified using the QIAquick Nucleotide Removal Kit cat. no. 28306 from Qiagen. Following osmium tagging (osmylation, see below), in-house HPLC analysis was used to determine the probe content, extent of osmylation, and efficiency of probe/target hybridization [31]. Osmylated oligos are stable and can be kept at −20 °C for 2 years. LoBind Eppendorf test tubes (1.5 mL) were used for serial 5/1000 or 10/1000 dilutions to yield probes at concentrations at about 40 fM. Mixtures of aliquots from the probe and from the isolated RNA were prepared in 0.5 mL RNase- and DNase-free sterile test tubes and used after the addition of 75 μL of ONT buffer.

The probes were developed and optimized earlier [29,31]. They are oligodeoxynucleotides with a sequence complementary to the target miRNA but extended at one end with five adjacent T residues and flanked by up to five adenosines (A) at either end (Table 2). The A-tails facilitated the entry of the probe into the nanopores. The adjacent Ts were each tagged with an osmium label, i.e., osmylated [38,39,40,41,42], with a 1:1 mixture of OsO_4_ and 2,2′-bipyridine (abbreviated OsBp) to slow down their translocation via the nanopores and distinguish them from native nucleic acids. Within the probe sequence complementary to the target, Ts were replaced by uridine (U), 2’-OMe-U or dU to minimize OsBp labeling because the osmylation kinetics of U and cytosine (C) are substantially slower than that of T [39,40,41,42]. HPLC analysis yields the probe’s concentration (content) using intact oligo as a standard because the absorbance of the probe at 260 nm is practically the same as that of the precursor intact oligo. HPLC analysis provided evidence of the quantitative depletion of the OsBp reagent which elutes ahead of the osmylated oligo.

**Table 2:** Sequence and characterization of the probes used in this work.

Probe ID	mU is 2’-OMe and dU is 2’-deoxyU; the Partial Sequence Between the A5 and T5 Is Complementary to the Target miRNA. All Five Ts Carry an OsBp tag.	Concentr., fM	No of OsBp, ^1^ Average
Probe15b	(A)_6_dUGdUAAACCAdUGAdUGdUGCdUGCdUA(T)_5_(A)_6_	43.4	5.9
Probe21	(A)_5_dUCAACAdUCAGdUCdUGAdUAAGCdUA(T)_5_C(A)_6_	36.6	6.2
Probe375	(A)_5_dUCACGCGAGCCGAACGAACAAAC(T)_5_C(A)_5_	41.6	5.9
Probe141	(A)_4_CCAmUCmUmUmUACCAGACAGmUGmUmUA(T)_5_(A)_5_	39.0	6.3

^1^ The average number of osmium label moieties on the probe (extent of osmylation) was measured using the following equation: absorbance at 312 nm/absorbance at 272 nm or R(312/272) = 2x(no osmylated pyrimidines/total nt) [39]. R is the ratio of the corresponding HPLC peaks, regardless of their shape (sharp or broad). An extra osmium tag was conjugated to a C or U base within the sequence. A single internal tag did not prevent hybridization, as shown by nanopore experiments.

### 4.3. Sample Preparation

This technology determines miRNA copy number by bracketing. At least two measurements are necessary and typically more than two are performed. The spread between the measurements determines the accuracy; for technical reasons (pipetting, oligo purity, etc.), accuracy at ±15% is a lower limit. Probe copies matter and should be kept around 100,000, somewhat more when the flow cell is new and somewhat less when the flow cell is old. The expected free probe copies should be detectable over the device’s noise observed at (*Ir*/*Io*)_max_ = 0.15. A spreadsheet may be used to simultaneously calculate the amount of total RNA sample and probe to target miR-15b at 1.56 HL (Table 3). If the Yenos test (4 experiments, see later) judges detection, then probe > target, and if silencing then probe < target or the equivalent target < 1.56 HL or target > 1.56 HL, respectively.

### 4.4. Single-Molecule Ion Channel Conductance Experiments on the MinION (MinION Mk1B Platform)

Registration is required at the ONT website to download the software MINKNOW to a computer/laptop with specifications provided by ONT. All the functions necessary to test the hardware and flow cells and run the experiments were performed using MINKNOW software. The sample was loaded onto a flow cell fitted in a MinION device. (Loading the sample without introducing air into the nanopore array is challenging and needs to be practiced on a used flow cell. Bubbles are impossible to remove from the flow cell’s microfluidics. Loading for sensing is different than loading for sequencing. The sample is loaded via the sample port and the excess solution is removed from the priming port. The experiment was run under “start sequencing” mode. A direct RNA sequencing kit (SQK-RNA002) was selected to initiate experiments. The flow cell type FLO-MIN106 was selected, even though flow cells are of version R10.4.1, SKU: FLO-MIN114. Run length (45 min) and bias voltage (−180 mV) were selected, base calling was disabled, and the output bulk file Raw (1–512) was checked and generated. The output location was Library/MinKNOW/data/, and the output format was *fast-5*. All the experiments reported here were run for 45 min at −180 mV. This type of sensing experiment produces less than a million of selected (by our algorithm) events in 45 min. A total of 45 min was used to permit the use of Microsoft Excel which limits the reported rows to about a million entries. The *fast-5* file was analyzed using the *OsBp_detect* algorithm [30]. The number of events per channel from the *OsBp_detect* analysis has been compared with the actual *I-t* trace of the specific channel using MATLAB (vs R2024b from Mathworks) visualization, and this algorithm, 2nd revision, has been repeatedly validated. Currently, *OsBp_detect* can only be used with a 2017 or earlier version of MacBook Pro loaded with macOS 10.14 Mojave. While alternative parameters were explored earlier, all experiments reported here were analyzed using the following threshold parameters: (i) event duration (in tps): 4–1200 (1.3–400 ms), (ii) lowest *I_r_*/*I_o_* < 0.55, and (iii) all *I_r_*/*I_o_* < 0.6, channels 1–512.

### 4.5. Data Analysis

A laptop/computer requires a few min for the *OsBp_detect* analysis of the *fast-5* file and produces a file in *tsv* format to be opened via Microsoft Excel and saved as such. In the Excel spreadsheet, the algorithm-selected events (*I_r_*/*I_o_* data) are grouped in the form of a histogram with 0.05 bins, from 0.05 to 0.55, and plotted (Supplementary Materials, pages 3–21). Histograms exhibit two maxima (*I_r_*/*I_o_*)*_max_*: an early one at *I_r_*/*I_o_* = 0.15 and a late one at *I_r_*/*I_o_* = 0.30. The Yenos probes translocate preferentially at the early (*I_r_*/*I_o_*)*_max_* = 0.15 [32]. These maxima may vary by ±0.05 units depending on the flow cell age. The events under late (*I_r_*/*I_o_*)*_max_* and early (*I_r_*/*I_o_*)*_max_* were noted, and their *ratio R* = ((*I_r_*/*I_o_*)*_max_ late *(0.3)/*early* (0.15)) was calculated. The R value represents the criterion by which an experiment is judged as detection or silencing compared to the buffer control. The number of events decreases with flow cell use, but R remains comparable in the absence of the free probe. In comparing a sample to buffer run, a decreasing *ratio* R indicates detection (free probe present), and a comparable or increasing *ratio* R indicates silencing (no free probe present) [32]. Comparison of R values is deemed significant at more than 18% change. The 18% change is not set in stone, and confirmation can be performed by running the sample again (see “nonew2” or 3rd run in the Supplementary Materials, e.g., page 11 (top left), page 19 (3rd on the right) and many examples on page 20). This assignment is consistent with an increased number of events owing to the presence of the probe, which traverses with (*I_r_*/*I_o_*)*_max_*~0.15, whereas intact RNA and background noise traverse mostly with (*I_r_*/*I_o_*)*_max_*~0.30. In the final optimized protocol, the buffer was run twice, the sample was run twice, and R values are calculated for each. Buffer and sample data may be added and reported as 1.5 h experiments (see Supplementary Materials, pages 3–20).

Because the flow cells lost active pores during every experiment, the *total* number of events decreases constantly with use. More than 200 active pores yield reliable data. Data analysis was optimized by deleting all channels which report more than 20,000 events. Typically, these channels represent less than 5%. Reasons to reject a test are when (i) one sample run exhibited an increasing R value and the other sample run exhibited a decreasing R value compared to the buffer R value, (ii) the (*I_r_*/*I_o_*)*_max_* shifted by more than ±0.05 units, or (iii) the sample produced events in large excess over the events produced by the buffer (see page 21 in Supplementary Materials).

## 5. Conclusions

Until recently, sample variability has been a major hurdle in the miRNA field, prohibiting the exploitation of miRNAs as cancer/disease biomarkers in liquid biopsies. Using the novel nanopore array-based MinION/Yenos platform, we have reported comparable miRNA copy numbers for four tested miRNAs from healthy subjects of distinct age, sex, and ethnicity. Comparable miRNA copy numbers were also determined from patients with breast, pancreatic, and prostate cancers, and they were about 1.8-fold elevated compared to the healthy subjects. Furthermore, comparable miRNA copies have been reported from serum or urine, suggesting that a urine sample may replace blood draw [32].

This study examined expression of selected miRNAs in a cohort of 22 cancer patients who provided serum samples, 2 healthy individuals who provided urine samples, and the commercially available combined serum of healthy men. It was determined that the levels of miR-15b, miR-21, miR-375, and miR-141 were comparable between the combined serum and the two urine samples and that they were all below the 1.5 HL threshold. In contrast, miR-21, miR-375, and miR-141 measured above the threshold of the 1.5-fold healthy level in the serum of treatment-naïve patients with breast, ovarian, prostate, pancreatic, lung, and colorectal cancer, while miR-15b measured below the 1.5-fold threshold. These finding are consistent with the notion that miR-21, miR-375, and miR-141 are cancer biomarkers, while miR-15b is not a cancer biomarker. The data are also consistent with the proposition that miRNA copy numbers are independent of subject’s age, sex, and ethnicity and that a urine sample may replace a blood draw for miRNA measurements.

An optimal experimental protocol and data analysis were proposed which may yield up to 97% sensitivity and 97% specificity for the above six cancer indications. If future work confirms the 97% accuracy in a larger sample size, then testing a subject by conducting three MinION/Yenos tests at the 1.5 HL threshold can be achieved with a single flow cell and should yield the “false” assignment in about 30 subjects in a million. Considering that our studies included treatment-naïve subjects, it is plausible that this test, as described here, may be used as a companion to facilitate doctor/patient decisions when the current tests for breast, prostate, pancreatic, ovarian, colorectal, and lung cancer fall into the “grey area”. For example, two such tests may be sufficient as a companion to a mammogram or a prostate test. Implementation in the general asymptomatic population will need to wait until there is evidence that miRNA cancer biomarkers are overexpressed years ahead of any symptoms. The latter study could be conducted using the Prostate, Lung, Colorectal, and Ovarian (PLCO) samples collected by the National Cancer Institute (NCI) from asymptomatic volunteers who developed cancer years after blood draw.

In our opinion, the single most important conjecture from this line of work is the observed independence of miRNA copy numbers in relation to biospecimens and a subject’s demographics. Support for the latter will revolutionize the miRNA field, yield reliable cancer screening tests, facilitate the development of miRNA therapeutics, and have a major impact on the early diagnosis of cancer.

## 6. Patents

(A. Kanavarioti, sole inventor)

(a) Osmium tagged probes for nucleic acid detection. Patent number: 11884968, granted 30 January 2024. (b) and (c) Nanopore platform for DNA/RNA oligo detection using an osmium tagged complementary probe. Patent number: 11427859, granted 30 August 2022, and patent number: 11111527, granted 7 September 2021. (d) International patents filed in 2023 in Europe, Canada, Australia, Japan, China, India, Brazil, and South Korea. (e) A provisional patent application entitled Detection, Quantification and Validation of microRNA Biomarkers was filed with the USPTO on 7 May 2024.

## Figures and Tables

**Table 1:** The results of a single Yenos test targeting the 1.5 HL level with each of the four miRNAs. Expectations: (i) miR-15b copies < 1.5 HL in all samples; miR-21, miR-375, and miR-141 > 1.5 HL in cancer samples but <1.5 HL in healthy samples. Measurement meeting expectation (ν), not meeting expectation (X), and not determined (ND).

Cancer Serum	ID	miR-15b	miR-21	miR-375	miR-141
lung	A1	ν	ν	ν	ν
ovarian	A2	ν	ν	ν	ν
“	A3	ν	ν	ν	ν
“	A4	ν	ν	ν	ν
“	A5	ν	ν	ν	ν
CRC ^1^	No1	ν	ν	ν	ν, ν
lung	No2	Ν, ν	X	ν, ν	ν, ν
CRC ^1^	No3	ν	ND	ν	ν, ν, ν
lung	No4	ν, ν	ν	ν, ν	ν, ν, ν
lung	No6	ν	ν, ν	X	ν, ν, ν
CRC ^1^	No7	ν	ν, ν	ν	ν, ν, ν
prostate	D1	ν	ν	ν	X
pancreatic	D2	ν	ν	X	ν
breast	D3	X	ν	ND	ν
“	D4	ν	ν	ν	ν
prostate	D5	X	ν	ν	ND
“	D6	ν	X	ν	ν, ν
“	D7	ν	ν	ν	ν
pancreatic	D8	ν	ν	ν	ν
prostate	D9	ν	ν	ν	ν
breast	D10	ν	ν	X	ν
pancreatic	D11	ν	ν	X	ν
**Healthy** **serum**	SQ H6914	ν, ν, ν	ν, ν, ν	ν, ν	ν, ν
	H6914 2025	ν	ν, ν	ν	ND
**Healthy** **urine**	Healthy1	ND	ν, ν	ND	ν, ν
	Healthy2	ν, ν	ν, ν	X	ν
					
**Score**		28/30	30/32	22/27	36/37
**% True ^2^**		93.3	93.8	81.5	97.3

^1^ CRC stands for colorectal. ^2^ % True for all 4 miRNAs with the 22 cancer samples standing at 0.912 and % True = 0.958 with the 4 healthy samples.

**Table 3:** **Example:** Sample preparation to detect miR-15b at the 1.56 HL in a sample of total RNA at 12.1 ng/μL using 4 mL of 43.4 fM probe15b.

Sample Volume in μL	5 μL		
Probe15b copies	Probe15b copies per 1 μL sample	Probe15b copies per 1 μL sample normalized to reference at 16 ng/μL	Probe15b copies per 1 μL sample normalized to the reference at 16 ng/μL and adjusted to target 1.56 HL miR-15b at 17,710 copies per 1 μL RNA from H6914 reference
=4 × 43.4 × 600	=4 × 43.4 × 600/5	=4 × 43.4 × 600/5 × 16/12.1	=4 × 43.4 × 600/5 × 16/12.1/17,710 = 1.56

## Data Availability

The data generated during this study are included in this published article. Raw data (fast5 format at ~3.3 GB each) may be obtained from the corresponding author.

## Supplementary Materials

The following supporting information can be downloaded at https://www.mdpi.com/article/10.3390/ijms26083822/s1.
